# Microbiological and host-derived biomarker evaluation following non-surgical periodontal therapy with short-term administration of systemic antimicrobials: secondary outcomes of an RCT

**DOI:** 10.1038/s41598-020-73054-8

**Published:** 2020-10-01

**Authors:** Raluca Cosgarea, S. Eick, S. Jepsen, N. B. Arweiler, R. Juncar, R. Tristiu, G. E. Salvi, C. Heumann, A. Sculean

**Affiliations:** 1 Department of Periodontology, Operative and Preventive Dentistry, University of Bonn, Welschnonnen str 17, 53111 Bonn, Germany; 2 Clinic for Prosthetic Dentistry, University Iuliu-Hatieganu, Cluj-Napoca, Romania; 3 Department of Periodontology and Peri-implant Diseases, Philipps University Marburg, Marburg, Germany; 4 Department of Periodontology, University of Bern, Bern, Switzerland; 5 Department of Dental Medicine, University of Oradea, Oradea, Romania; 6 Department for Statistics, Ludwig-Maximilians University, Munich, Germany

**Keywords:** Health care, Dentistry, Periodontics, Periodontitis, Diseases, Dental diseases

## Abstract

Nonsurgical periodontal therapy with adjunctive use of systemic antimicrobials (for 7–14 days) showed improved clinical, microbiological and immunological results over the mechanical protocol alone. Considering the increasing risk for antimicrobial resistance with longer antibiotic regimes, it is important to establish the optimal antibiotic protocol with a maximum antimicrobial benefit and minimum risk for adverse effects. The aim of the study was to evaluate the microbiological and inflammatory outcomes 12-months after a 3-/7-day systemic antibiotic protocol [amoxicillin (AMX) + metronidazole (MET)] adjunctive to subgingival debridement in severe periodontitis compared to mechanical treatment alone. From the initially treated 102 patients, 75 subjects (Placebo group: n = 26; 3-day AMX + MET group: n = 24; 7-day AMX + MET group: n = 25) completed the 12-month examination. Clinical parameters, eight periodontal pathogens and inflammatory markers were determined at baseline and 3-, 6-, 12-months after therapy using real-time PCR and ELISA respectively. After 6 months, several periodontopathogens were significantly more reduced in the two antibiotic groups compared to placebo (*p* < 0.05). After 1 year, both antibiotic protocols showed significant reductions and detection of the keystone pathogen *P. g*ingivalis compared to placebo. Antibiotic protocols, smoking, disease severity, baseline-BOP, -CAL and -IL-1β, as well as detection of *T. denticola* at 12-months significantly influenced the residual number of deep sites. The present data indicate that the systemic use of both short and longer antibiotic protocols (AMX + MET) adjunctive to nonsurgical periodontal therapy lead to higher microbiological improvements compared to subgingival debridement alone. The two investigated antibiotic protocols led to comparable microbiological and inflammatory results.

## Introduction

Since periodontitis is a biofilm-induced inflammatory multifactorial chronic disease, periodontal treatment aims at reducing the supra- and subgingival biofilm by institution of an adequate oral hygiene and meticulous debridement of the roots (i.e. subgingival debridement-SD) in order to resolve tissue inflammation and arrest disease progression^1^. Despite the fact that SD has been shown to be successful in reducing periodontal pathogens in the subgingival area^2,3^, evidence exists that major periodontal pathogens like *Aggregatibacter actinomycetemcomitans* or *Porphyromonas gingivalis* may not be completely eliminated by mechanical debridement alone and their persistence has been associated with further tissue breakdown^4–6^. Vice versa, the absence of certain periodontal pathogens has been associated with lower risk for further attachment-loss^7–9^. Consequently, systemic antimicrobials had been introduced in the late 80ies as adjuncts to mechanical debridement to reinforce SD and sustain the host-defence system by reaching the pathogens not always accessible for mechanical instruments (i.e. root-concavities, furcations) or in other mouth areas (i.e. pharynx, tongue)^10^.

In this sense, several antimicrobials and various combinations thereof had been implemented in the non-surgical periodontal treatment showing improved clinical, microbiological and immunological results over the mechanical protocol alone^11–15^. The use of a combination of amoxicillin (AMX) and metronidazole (MET) dominates the literature in this topic^14–16^, indicating a synergistic efficiency against gram-negative anaerobes in particular^17–21^. Statistically significantly better clinical outcomes regarding probing pocket depth (PD) reduction and clinical attachment level (CAL) gain^11,13,22–33^ as well as significant reduction of periodontal pathogens and of inflammatory cytokines were observed for the systemic use of AMX + MET adjunctive to SD compared to mechanical treatment alone^22–32,34,35^. However, it is important to emphasize that for initially shallow (PD < 4 mm) and moderate (PD 4–5 mm) sites in patients with chronic periodontitis, the adjunctive use of AMX + MET has been shown to have only minimal additional clinical benefits (PD reduction and CAL gain)^13,36^. On the other hand, initially deep sites (PD > 6 mm) have demonstrated substantial additional clinical improvements (i.e. 0.74 mm PD reduction and 0.61 mm CAL gain after 12 months)^13^, thus decreasing significantly the need for subsequent surgical periodontal therapy^22,25,30,37^. Moreover, it has been shown that patients taking the antimicrobials at the initial phase of the periodontal treatment, exhibited significantly greater clinical improvements in initially deep sites compared to those taking the antimicrobials after healing or at the second stage^25,37^.

Despite the abundance of studies on AMX + MET in non-surgical periodontal therapy, there seems to be no consensus regarding the optimal dosage and duration of the medication: doses of each of the antimicrobials range from 200 to 500 mg for durations of 3–14 days^20–30,32,33,38–41^. In the light of an increasing risk for antimicrobial resistance with longer antibiotic regimes, it is important to establish the optimal antibiotic protocol with a maximum antimicrobial benefit and minimum risk for adverse events (i.e. antibiotic resistance, hypersensitivity, renal/liver toxicity, e.t.c). This has to consider the pharmacological principle that antimicrobials should be taken in a minimum bactericidal concentration for a minimum duration^42^ and is in concordance with the current worldwide concern regarding the critical levels of antibiotic resistance as a result of the indiscriminative use of antimicrobials^12,43,44^. Since the discovery of antimicrobials in the 1940s, the abundance of antibiotic-resistant genes has significantly increased^45^. Evidence also indicates that oral microbiota represent a reservoir for transferable antimicrobial resistant genes^46–49^ having the potential to transfer antimicrobial resistance in patients undergoing antibiotic therapy as well^50^. Thus, it seems mandatory, that not only in various field of general medicine the antibiotic prescription and protocol should be reanalysed and clearly defined^51^, but also in dentistry and especially in periodontal therapy there is an urgent need to establish the optimal antibiotic protocol and the class of patients that may really benefit from it.

Studies evaluating the clinical, microbiological and immunological efficacy of a short-term administration of AMX + MET compared to the standard protocol (e.g. use for at least 7 days) in non-surgical periodontal therapy are scarce. Lately, we have evaluated the clinical outcomes at 6 and 12 months of a 3-day regimen of AMX + MET adjunctive to SD in severe chronic periodontitis showing that both antibiotic protocols (3 and 7 days) led to statistically significant better clinical improvements compared to SD alone^33,41^. The aim of the present analysis, was therefore to evaluate the microbiological and inflammatory biomarkers outcomes following non-surgical periodontal therapy in conjunction with systemic administration of AMX + MET for 3 or 7 days in patients with severe chronic periodontitis (stage III–IV, grade B periodontitis). The present article represents the microbiological and inflammatory analyses of a previous RCT^33,41^.

## Results

### Patients

Hundred two subjects (mean age 43.37 ± 9.85, 65 female, 35 smokers, n = 34/group) were enrolled in the study and 27 patients dropped-out at the 12-month evaluation. Reasons for exclusion from the final analysis were antibiotic intake for other medical reasons, non-compliance with the appointments schedule and moved out of town. No statistically significant differences (*p* > 0.05) between the groups were detectable at baseline (i.e. for gender distribution, smoking status, clinical parameters: PD, CAL, BOP, FMPS, number of deep sites with PD ≥ 6 mm, e.t.c.) (Table 1 in Cosgarea et al.)^33^. Excellent patient compliance with the pills intake and only minor adverse events were registered in all three treatment groups^33^. The main outcomes at 12 months indicated statistically significantly better clinical improvements (i.e. PD-reduction, CAL-gain) for the 7-day antimicrobial-protocol as compared to placebo, while statistically significantly fewer residual deep sites (PD ≥ 6 mm) were present in the 3-day Antimicrobial-group as compared to placebo^41^. Additionally, statistically significantly more patients reached a periodontal status with low risk for disease progression (≤ 4 sites with PD ≥ 5 mm) in the two antimicrobial-groups as compared to placebo^41^.

**Table 1:** Detection of *A. actinomycetemcomitans* and *P. gingivalis*. Detection of the periodontopathogens at baseline, 3, 6 and 12 months (number of subjects) in patients positive/negative before treatment foe each treatment group: Group A: Placebo, Group B: 3 days AMX + MET, Group C: 7 days AMX + MET (*Aa: A. actinomycetemcomitans; Pg: P. gingivalis*; m: months; Base: baseline; m: months; N: number of patients; “ + ”: subjects positive for the bacterium; “−” subjects negative for the bacterium).

Microorganism	*A. actinomycetemcomitans*			*P. gingivalis*		
Timepoints	Group A N = 26	Group B N = 24	Group C N = 25	Group A N = 26	Group B N = 24	Group C N = 25
**Subjects positive at base**						
Base	15	7	11	29	26	28
3 m+	11	2	4	18	11	6
3 m−	4	5	7	11	15	22
6 m+	9	4	5	19	12	6
6 m−	6	3	6	10	14	22
12 m+	10	3	7	24	14	14
12 m−	5	4	4	5	12	14
**Subjects negative at base**						
Base	15	23	20	1	4	3
3 m+	4	1	6	1	1	1
3 m−	11	22	14	0	3	2
6 m+	3	6	5	1	1	1
6 m−	12	17	15	0	3	2
12 m+	4	6	8	1	2	0
12 m−	11	17	12	0	2	3

### Microbiological results

Detection of the main periodontal pathogens *A. actinomycetemcomitans* and *P. gingivalis* as related to their presence/absence prior treatment are presented in Table 1. *A. actinomycetemcomitans* was detectable at 6 and 12 months in a higher number of patients in the placebo group as opposed to the antimicrobial-groups (group B and C); moreover, antimicrobials were not able to completely eradicate (i.e. below the detection limit) this periodontal pathogen in neither of the antimicrobial-groups. Similar observations were seen for *P. gingivalis* (Table 1).

Nonetheless, patients initially positive on *P. gingivalis* showed at 6 months statistically significantly fewer residual deep sites (PD ≥ 6 mm) in the two antimicrobial-groups compared to the placebo group (*p* < 0.05); this was maintained up to 12 months in the 3-day antimicrobial-group (*p* < 0.05) (Table 2). Significantly more treated sites resulted in pocket closure (PD < 4 mm) at 6 months in the two antimicrobial-groups compared to placebo (*p* < 0.05, Table 2). The 3-day antimicrobial-group showed significantly more (*p* = 0.044) healed sites compared to placebo also at 12 months. However, no statistically significant differences were obtained between the two groups receiving antimicrobials (*p* > 0.05). Only patients positive on *P. gingivalis* at 6 months had statistically significantly more deep sites in the placebo group as opposed to the 3-day antimicrobial-group (*p* = 0.001).

**Table 2:** Outcomes for residual deep sites and healed sites depending on the presence of *A. actinomycetemcomitans*, *P. gingivalis* and *T. denticola*. Mean number (N) of residual deep sites (PD ≥ 6 mm) and of sites achieving pocket closure (PD < 4 mm) at 6 and 12 m depending on the presence/absence at baseline, at 6 or 12 months of the periodontal pathogens *A. actinomycetemcomitans*, *P. gingivalis* and *T. denticola* (Group A: Placebo, Group B: 3 days AMX + MET, Group C: 7 days AMX + MET; *Aa: A. actinomycetemcomitans; Pg: P. gingivalis*; m: months; Base: baseline; N: number of patients). Baseline, 6 m and 12 m data for the number of sites ≥ 6 mm of all patients had been previously published in Cosgarea et al. 2017.

Variables	Group A (N = 26) Mean ± SD		Group B (N = 24) Mean ± SD		Group C (N = 25) Mean ± SD		*p* (A–B)		*p* (A–C)		*p* (B–C)	
	PD ≥ 6 mm	PD ≤ 3 mm	PD ≥ 6 mm	PD ≤ 3 mm	PD ≥ 6 mm	PD ≤ 3 mm	PD ≥ 6 mm	PD ≤ 3 mm	PD ≥ 6 mm	PD ≤ 3 mm	PD ≥ 6 mm	PD ≤ 3 mm
**All patients**	N = 26	N = 26	N = 24	N = 24	N = 25	N = 25						
Base	25.50 ± 17.36	–	29.37 ± 14.65	–	35.92 ± 17.58	–	1.000	–	0.098	–	0.592	–
6 m	8.92 ± 6.63	38.70 ± 19.10	2.08 ± 3.43	52.60 ± 19.60	5.00 ± 5.11	51.90 ± 19.00	< 0.001	0.039	0.001	0.051	0.303	1.000
12 m	5.19 ± 3.71	39.00 ± 17.30	1.66 ± 2.26	52.30 ± 19.20	4.52 ± 5.49	51.50 ± 19.90	0.003	0.044	0.917	0.059	0.068	1.000
**Aa negative (Base)**	N = 12	N = 12	N = 19	N = 19	N = 16	N = 16						
6 m	6.83 ± 5.54	39.17 ± 20.92	2.53 ± 3.75	53.32 ± 18.45	4.94 ± 5.43	52.19 ± 19.17	0.005	0.160	0.086	0.254	0.924	1.000
12 m	4.92 ± 3.55	39.25 ± 17.75	1.47 ± 1.98	54.11 ± 18.3	4.06 ± 5.42	53.25 ± 21.12	0.019	0.124	0.664	0.187	0.302	1.000
**Aa positive (Base)**	N = 14	N = 14	N = 5	N = 5	N = 9	N = 9						
6 m	9.57 ± 6.65	38.4 ± 18.3	4.00 ± 5.48	50.0 ± 26.11	4.33 ± 3.12	51.4 ± 19.9	0.008	0.840	0.047	0.428	0.886	1.000
12 m	4.71 ± 3.63	38.71 ± 1.758	2.00 ± 2.92	45.20 ± 23.18	4.56 ± 4.39	48.44 ± 18.2	0.603	1.000	1.000	0.709	0.924	1.000
**Aa negative**												
At 6 m	7.94 ± 5.43	41.11 ± 21.44	2.31 ± 3.83	54 ± 10 ± 20.26	4.65 ± 5.34	52.51 ± 19.55	0.001	0.200	0.037	0.345	0.933	1.000
At 12 m	4.83 ± 3.47	41.30 ± 17.63	1.64 ± 2.22	51.51 ± 19.35	3.78 ± 5.07	56.22 ± 19.51	0.015	0283	0.487	0.065	0.560	1.000
**Aa positive**												
At 6 m	8.90 ± 7.42	35.00 ± 15.09	1.20 ± 8.37	47.21 ± 18.17	4.88 ± 3.09	50.81 ± 19.07	0.005	0.630	0.030	0.204	1.000	1.000
At 12 m	4.75 ± 3.88	33.81 ± 16.41	1.00 ± 1.41	60.51 ± 21.92	5.43 ± 4.93	39.61 ± 16.39	0.355	0.283	1.000	0.065	0.303	1.000
**Pg negative (Base)**	N = 1	N = 1	N = 4	N = 4	N = 3	N = 3						
6 m	7.00	12.00	0 ± 0	59.0 ± 20.69	3.33 ± 3.51	41.67 ± 20.31	0.196	0.288	0.840	0.799	0.549	0.958
12 m	6.00	18.00	0 ± 0	55.0 ± 18.89	4.67 ± 7.23	44.00 ± 30.51	1.000	0.690	1.000	1.000	0.774	1.000
**Pg positive (Base)**	N = 25	N = 25	N = 20	N = 20	N = 22	N = 22						
6 m	8.36 ± 6.32	39.80 ± 18.74	2.50 ± 3.63	51.35 ± 19.75	4.91 ± 4.84	53.32 ± 18.89	< 0.001	0.144	0.003	0.055	0.748	1.000
12 m	4.76 ± 3.59	39.80 ± 17.11	1.90 ± 2.22	51.70 ± 19.71	4.18 ± 4.84	52.55 ± 18.79	0.013	0.106	0.700	0.064	0.272	1.000
**Pg negative**												
At 6 m	9.67 ± 6.91	49.71 ± 19.87	2.20 ± 3.49	54.40 ± 19.15	4.71 ± 4.95	53.21 ± 19.89	0.003	x	0.028	x	0.771	
At 12 m	3.50 ± 3.59	38.51 ± 14.77	1.00 ± 1.69	53.90 ± 19.75	3.89 ± 4.83	53.80 ± 21.59	0.376	0.255	1.000	0.226	0.186	1.000
**Pg positive**												
At 6 m	7.59 ± 5.88	32.90 ± 16.51	1.89 ± 3.55	49.70 ± 21.31	4.75 ± 3.30	45.01 ± 13.39	0.001	1.000	0.153	1.000	1.000	1.000
At 12 m	5.39 ± 3.43	39.21 ± 18.72	2.56 ± 2.55	49.01 ± 19.12	5.33 ± 5.78	44.30 ± 11.60	0.095	0.492	1.000	1.000	0.556	1.000
**Td negative (Base)**	N = 3	N = 3	N = 0	N = 0	N = 1	N = 1						
6 m	9.00 ± 8.18	36.01 ± 20.88	–	–	0.00 ± 0.00	32.02 ± 0.00	–	–	0.527	0.883	–	–
12 m	5.33 ± 2.01	38.71 ± 24.68	–	–	0.00 ± 0.00	35.00 ± 0.00	–	–	0.412	0.909	–	–
**Td positive (Base)**	N = 23	N = 23	N = 24	N = 24	N = 24	N = 24						
6 m	8.22 ± 6.13	39.10 ± 19.39	2.08 ± 3.44	52.60 ± 19.66	4.92 ± 4.66	52.81 ± 18.96	< 0.001	0.058	0.001	0.054	0.499	1.000
12 m	4.74 ± 5.01	39.01 ± 16.88	1.58 ± 2.15	52.31 ± 19.21	4.42 ± 5.01	52.20 ± 19.98	0.008	0.055	1.000	0.056	0.092	1.000
**Td negative**												
At 6 m	7.60 ± 7.04	43.20 ± 18.92	2.07 ± 3.55	55.55 ± 21.71	5.20 ± 4.90	49.41 ± 19.26	0.017	0.448	0.262	1.000	0.421	1.000
At 12 m	3.43 ± 3.18	44.03 ± 18.88	1.00 ± 1.69	56.61 ± 19.04	2.94 ± 4.25	55.30 ± 21.88	0.080	0.305	1.000	0.392	0.524	1.000
**Td positive**												
At 6 m	8.75 ± 5.81	35.91 ± 19.36	2.09 ± 3.48	49.22 ± 17.31	2.80 ± 3.27	62.00 ± 15.76	< 0.001	0.220	0.004	0.027	1.000	0.606
At 12 m	6.42 ± 3.32	33.10 ± 13.75	2.56 ± 2.55	45.21 ± 18.38	6.56 ± 5.59	44.81 ± 14.36	0.050	0.257	1.000	0.292	0.225	1.000

Both initially negative and positive patients for *A. actinomycetemcomitans* showed at 6 months statistically significantly less deep sites in the two antimicrobial-groups compared to placebo. Nonetheless, only those initially negative had at 12 months still significantly less deep sites in the 3-day -group compared to placebo (Table 2). Similar findings were observed for the patients negative for *A. actinomycetemcomitans* at 6/12 months.

Patients initially negative on *T. denticola* had significantly less deep sites at 6 and 12 months in the 3-day antimicrobial-group compared to placebo, while those in the 7-day antimicrobial-group showed only at 6 months significantly less such sites. Subjects positive on *T. denticola* at 6 months had in both antimicrobial-groups significantly less deep sites than placebo, while at those positive at 12 months showed a borderline significance (*p* = 0.05) for the 3-day antimicrobial-group (Table 2).

Initial presence or absence (prior treatment) or at the follow-ups of *A. actinomycetemcomitans*, *P. gingivalis* or *T. denticola* did not have any influence on the number of sites reaching pocket closure neither at 6 nor at 12 months (Table 2).

At 12 months compared to baseline, quantitative microbial analyses showed statistically significant reductions in the proportions of *P. gingivalis, T. forsythia, C. rectus* and *F. allocis* in all treatment groups (Table 3)*. T. denticola* was statistically significantly reduced at 12 months compared to baseline only in the antimicrobial-groups, while *A. actinomycetemcomitans* was reduced only in the 7-day antimicrobial-group and only at the 6 months evaluation; at 12 months, there were no significant reductions compared to baseline in none of the groups (*p* > 0.05). Nonetheless, group comparisons revealed no significant quantitative differences at baseline for neither of the microorganisms, with the exception of *A. actinomycetemcomitans*, which was present in significantly higher quantities in the placebo compared to the 3-day group (Table 3). Following, at 12 months, statistically significantly higher reductions in the mean counts of the *P. gingivalis* were seen in both antimicrobial-groups compared to placebo. *F. nucleatum* and *F. allocis* were statistically significantly reduced only in the 7-day AB group compared to placebo. Neither at 6 nor at 12 months, no statistically significant differences were seen between the two antibiotic groups for any of the microorganisms (*p* > 0.05, Table 2).

**Table 3:** Quantitative microbiological results. Mean values and group comparisons (Mann Whitney U for inter-group comparisons, Wilcoxon Signed Ranks test) for intra-group comparisons between various timepoints) for microbiological parameters (mean ± SD) (Group A: Placebo, Group B: 3 days AMX + MET, Group C: 7 days AMX + MET; ^s^statistical significant *p* values 0.001 < *p* < 0.05; ^hs^highly statistically significant values *p* ≤ 0.001; Base: baseline, m: months, Δ: change).

Variables	Group A N = 26	Group B N = 24	Group C N = 25	*p* value Mann–Whitney A–B	*p* value Mann–Whitney A–C	*p* value Mann–Whitney B–C
***A. actinomycetemecomitans (× 10***^**5**^**)**						
Base	0.70 ± 2.11	0.11 ± 0.54	1.92 ± 6.29	0.036^s^	0.471	0.204
3 m	0.37 ± 1.06	0.01 ± 0.05^ s^	1.86 ± 9.30	0.001^hs^	0.053	0.213
6 m	0.74 ± 2.76	0.02 ± 0.08	0.15 ± 0.73^s^	0.138	0.244	0.775
12 m	0.17 ± 0.48	0.74 ± 3.65	1.10 ± 4.79	0.104	0.961	0.098
Δ base-3 m	0.35 ± 2.3	0.10 ± 0.48	0.34 ± 5.90	0.652	0.624	0.839
Δ base-6 m	− 0.015 ± 1.56	0.10 ± 0.50	1.57 ± 5.77	0.971	0.617	0.672
Δ base-12 m	0.65 ± 2.18	− 0.61 ± 3.04	1.34 ± 8.63	0.738	0.600	0.640
Δ 3 m–6 m	− 0.38 ± 2.67	− 0.004 ± 0.01	1.76 ± 8.71	0.114	0.805	0.128
Δ 3 m–12 m	− 0.071 ± 0.41	− 0.73 ± 3.59	1.14 ± 11.47	0.617	0.429	0.465
Δ 6 m–12 m	0.58 ± 2.88	− 0.76 ± 3.65	− 1.01 ± 5.05	0.806	0.317	0.289
***P. Gingivalis (× 10***^**6**^**)**						
Base	5.06 ± 10.85^hs^	4.65 ± 12.81^hs^	9.42 ± 26.45^hs^	0.124	0.971	0.266
3 months	0.33 ± 1.50^hs^	0.52 ± 2.09^s^	0.01 ± 0.07^hs^	0.232	< 0.0001^hs^	0.014s
6 months	0.31 ± 1.08^hs^	1.23 ± 5.92^hs^	0.001 ± 0.006^hs^	0.037^s^	< 0.0001^hs^	0.078
12 m	0.13 ± 0.25^hs^	0.07 ± 0.19^hs^	0.012 ± 0.035^hs^	0.033^s^	< 0.0001^hs^	0.210
Δ base-3 m	4.89 ± 10.84	4.13 ± 13.05	10.41 ± 28.26	0.158	0.902	0.155
Δ base-6 m	4.89 ± 10.90	3.80 ± 14.66	9.98 ± 27.29	0.142	0.613	0.054
Δ base-12 m	3.81 ± 6.51	4.06 ± 13.07	11.84 ± 29.75	0.149	0.509	0.060
Δ 3 m–6 m	0.022 ± 0.05	− 0.63 ± 5.90	0.01 ± 0.07	0.479	0.206	0.838
Δ 3 m–12 m	0.28 ± 1.62	0.58 ± 2.36	0.005 ± 0.09	0.204	0.312	0.479
Δ 6 m–12 m	0.19 ± 1.16	1.28 ± 6.32	− 0.01 ± 0.034	0.550	0.565	0.784
***T. denticola (× 10***^**6**^**)**						
Base	0.30 ± 0.57	0.23 ± 0.30	0.76 ± 1.81	0.367	0.207	0.634
3 m	0.052 ± 0.14^s^	0.01 ± 0.035^hs^	0.02 ± 0.05^hs^	0.085	0.081	0.694
6 m	0.05 ± 0.14^s^	0.06 ± 0.22^hs^	0.03 ± 0.08^hs^	0.047^s^	0.012^s^	0.421
12 m	0.16 ± 0.48	0.03 ± 0.07^hs^	0.007 ± 0.016^hs^	0.256	0.103	0.644
Δ base-3 m	0.25 ± 0.62	0.22 ± 0.30	0.85 ± 1.93	0.062	0.057	0.424
Δ base-6 m	0.25 ± 0.59	0.19 ± 0.39	0.78 ± 1.88	0.194	0.126	0.692
Δ base-12 m	0.058 ± 0.57	0.21 ± 0.29	0.94 ± 2.03	0.103	0.008^s^	0.149
Δ 3 m–6 m	− 0.006 ± 0.19	− 0.05 ± 0.19	− 0.16 ± 0.06	0.763	0.428	0.334
Δ 3 m–12 m	− 0.11 ± 0.53	− 0.02 ± 0.07	− 0.000 ± 0.03	0.658	0.727	0.277
Δ 6 m–12 m	− 0.089 ± 0.52	0.034 ± 0.009	0.009 ± 0.05	0.683	0.494	0.733
***T. forsythia (× 10***^**6**^**)**						
Base	6.13 ± 12.01	7.17 ± 10.41	11.90 ± 28.24	0.628	0.589	0.988
3 m	1.16 ± 2.85^s^	0.04 ± 0.16^hs^	0.027 ± 0.11^hs^	0.031^s^	0.046^s^	0.928
6 m	30.77 ± 160.26^s^	0.21 ± 0.60^hs^	0.98 ± 3.56^s^	0.040^s^	0.012^s^	0.499
12 m	0.42 ± 0.99^hs^	0.65 ± 2.43^s^	0.36 ± 1.38^hs^	0.432	0.674	0.238
Δ base-3 m	4.96 ± 12.82	7.13 ± 10.44	13.27 ± 30.08	0.307	0.195	0.686
Δ base-6 m	− 24.42 ± 161.92	6.79 ± 11.01	11.56 ± 29.76	0.422	0.234	0.646
Δ base-12 m	6.09 ± 13.02	6.08 ± 11.39	12.65 ± 31.60	0.918	0.402	0.417
Δ 3 m–6 m	− 30.98 ± 163.26	− 0.16 ± 0.49	− 1.07 ± 3.76	0.783	0.645	0.516
Δ 3 m–12 m	0.98 ± 3.31	− 0.60 ± 2.45	− 0.34 ± 1.49	0.213	0.785	0.248
Δ 6 m–12 m	38.37 ± 179.99s	− 0.44 ± 2.61	0.88 ± 4.43	0.075	0.111	0.774
***P. micra (× 10***^**6**^**)**						
Base	0.112 ± 0.254	0.308 ± 0.736	0.276 ± 0.780	0.271	0.367	0.937
3 m	0.180 ± 0.476	0.252 ± 1.032^s^	0.045 ± 0.137^hs^	0.052	0.003^s^	0.175
6 m	0.177 ± 0.380	0.127 ± 0.467^s^	0.031 ± 0.086^s^	0.021^s^	< 0.0001^hs^	0.215
12 m	0.311 ± 0.587	0.112 ± 0.272	0.206 ± 0.869^s^	0.601	0.244	0.435
Δ base-3 m	− 0.064 ± 0.55	0.05 ± 0.65	0.24 ± 0.85	0.168	0.074	0.678
Δ base-6 m	− 0.065 ± 0.45	0.14 ± 0.80	0.26 ± 0.81	0.009^s^	0.009^s^	0.987
Δ base-12 m	− 0.23 ± 0.57	0.25 ± 0.86	0.12 ± 1.26	0.146	0.020^s^	0.543
Δ 3 m–6 m	0.000 ± 0.59	0.16 ± 0.99	0.13 ± 0.15	0.525	0.425	0.827
Δ 3 m–12 m	− 0.22 ± 0.67	0.20 ± 1.21	− 0.22 ± 0.93	0.881	0.934	0.550
Δ 6 m–12 m	− 0.12 ± 0.72	0.026 ± 0.58	− 0.19 ± 0.92	0.474	0.262	0.899
***F. Nucleatum (× 10***^**6**^**)**						
Base	2.29 ± 1.66	3.18 ± 4.21	3.70 ± 4.06	0.918	0.341	0.554
3 m	1.29 ± 1.51^s^	0.75 ± 1.26^hs^	0.54 ± 0.74^hs^	0.040^s^	0.018^s^	0.923
6 m	2.12 ± 2.58	0.96 ± 1.36^hs^	0.51 ± 0.93^hs^	0.027^s^	< 0.0001^hs^	0.132
12 m	2.25 ± 2.08	9.44 ± 36.18	1.01 ± 1.40^hs^	0.516	0.011^s^	0.137
Δ base-3 m	1.02 ± 1.92	2.43 ± 4.11	3.32 ± 4.29	0.154	0.031^s^	0.330
Δ base-6 m	0.13 ± 3.11	2.42 ± 4.39	3.04 ± 4.33	0.043^s^	0.009^s^	0.399
Δ base-12 m	0.25 ± 2.68	− 6.02 ± 37.11	3.45 ± 4.71	0.364	0.003^s^	0.080
Δ 3 m–6 m	− 0.93 ± 2.21	− 0.11 ± 0.82	0.02 ± 1.07	0.359	0.062	0.244
Δ 3 m–12 m	− 0.96 ± 1.87	− 8.55 ± 36.33	− 0.44 ± 1.16	0.670	0.159	0.509
Δ 6 m–12 m	− 0.21 ± 2.63	− 8.83 ± 37.15	− 0.55 ± 0.99	0.177	0.401	0.440
***C. Rectus (× 10***^**6**^**)**						
Base	0.42 ± 0.36	0.56 ± 0.58	0.84 ± 0.89	0.652	0.072	0.257
3 m	0.18 ± 0.27^s^	0.082 ± 0.13^hs^	0.067 ± 0.11^hs^	0.061	0.030^s^	0.658
6 m	0.31 ± 0.68	0.12 ± 0.24^hs^	0.082 ± 0.24^hs^	0.082	0.002^s^	0.099
12 m	0.21 ± 0.23^s^	9.46 ± 45.61^s^	0.15 ± 0.23^hs^	0.105	0.221	0.917
Δ base-3 m	0.23 ± 0.48	0.48 ± 0.59	0.84 ± 0.96	0.219	0.009^s^	0.157
Δ base-6 m	0.10 ± 0.77	0.47 ± 0.67	0.67 ± 0.83	0.194	0.009^s^	0.212
Δ base-12 m	0.20 ± 0.49	− 8.85 ± 45.68	0.79 ± 0.98	0.201	0.023^s^	0.375
Δ 3 m–6 m	− 0.13 ± 0.68	− 0.028 ± 0.18	− 0.017 ± 0.23	0.768	0.889	0.475
Δ 3 m–12 m	− 0.04 ± 0.24	− 9.37 ± 45.61	− 0.088 ± 0.25	0.806	0.404	0.522
Δ 6 m–12 m	0.15 ± 0.76	− 9.74 ± 46.58	− 0.10 ± 0.22	0.982	0.311	0.310
***F. Allocis (× 10***^**6**^**)**						
Base	1.60 ± 3.17	1.99 ± 3.52	2.49 ± 9.09	0.506	0.762	0.242
3 m	0.31 ± 0.76^hs^	0.058 ± 0.17^hs^	0.015 ± 0.063^hs^	0.002^s^	< 0.0001^hs^	0.762
6 m	0.27 ± 0.53^hs^	0.11 ± 0.25^hs^	0.033 ± 0.12^hs^	0.085	0.001^hs^	0.123
12 m	0.071 ± 0.18^hs^	0.026 ± 0.059^hs^	0.021 ± 0.077^hs^	0.553	0.025^s^	0.083
Δ base-3 m	1.32 ± 3.06	1.93 ± 3.55	2.66 ± 9.75	0.231	0.594	0.243
Δ base-6 m	1.38 ± 3.22	1.86 ± 3.75	2.56 ± 9.41	0.381	0.657	0.555
Δ base-12 m	1.64 ± 3.39	2.12 ± 3.81	2.95 ± 10.33	0.303	0.992	0.293
Δ 3 m–6 m	0.04 ± 0.78	− 0.05 ± 0.14	− 0.02 ± 0.14	0.128	0.415	0.179
Δ 3 m–12 m	0.28 ± 0.75	0.04 ± 0.16	− 0.004 ± 0.11	0.149	0.054	0.926
Δ 6 m–12 m	0.26 ± 0.60	0.10 ± 0.23	− 0.007 ± 0.026	0.270	0.013^s^	0.388

At 12 months statistically significantly, less patients were positive on *P. gingivalis* and *F. allocis* compared to baseline in the two antimicrobial-groups (Table 4), while *T. denticola* was less detectable in all treatment groups (*p* < 0.05). Detection of *A. actinomycetemcomitans* did not show any statistically significant reduction in any of the treatment groups neither at 6 nor at 12 months (*p* > 0.05, Table 4). Group comparisons revealed that even if at 6 months several bacteria (*P. gingivalis, T. denticola, T. forsythia, F. nucleatum, C. rectus, F. allocis*) were statistically significantly less detectable in the 7-day antimicrobial-group compared to placebo, at 12 months no significant differences were registered between the groups. Only *P. gingivalis* was significantly less detectable in both antimicrobial-groups compared to placebo while *F. allocis* only in the 7-day antimicrobial-group (Table 4).

**Table 4:** Qualitative microbial analysis. Detection frequency of periodontopathogenic microorganisms (Group A: Placebo, Group B: 3 days AMX + MET, Group C: 7 days AMX + MET; ^s^statistical significant *p* values 0.001 < *p* < 0.05; ^hs^highly statistically significant values *p* ≤ 0.001; Base: baseline, m: months, Δ: change).

Variables	Group A (n = 26) n/%	Group B (n = 24) n/%	Group C (n = 25) n/%	*p* (A–B)	*p* (A–C)	*p* (B–C)
***A. actinomycetemcomitans***						
Base	15/50	7/23.3	11/35.5	0.032^s^	0.259	0.305
3 m	15/50	3/10	10/32.3	0.001^s^	0.165	0.034s
6 m	12/40	10/33.3	10/32.3	0.599	0.537	0.930
12 m	14/46.7	9/30	15/48.4	0.190	0.895	0.146
***P. gingivalis***						
Base	29/96.7	26/86.7	28/90.3	0.168	0.325	0.661
3 m	19/63.3^s^	12/40^hs^	7/22.6^hs^	0.073	0.001^s^	0.147
6 m	20/66.7^s^	13/43.3^hs^	7/22.6^hs^	0.071	< 0.000^hs^	0.087
12 m	25/83.3	16/53.3^s^	14/54.2^hs^	0.012^s^	0.001^hs^	0.531
***T. denticola***						
Base	27/90	30/100	30/96.8	0.078	0.293	0.329
3 m	17/56.7^s^	12/40^hs^	12/38.7^hs^	0.203	0.166	0.920
6 m	20/66.7^s^	15/50^hs^	10/32.3^hs^	0.197	0.007^s^	0.164
12 m	19/63.3^s^	15/50^hs^	16/51.6^hs^	0.305	0.363	0.902
***T. forsythia***						
Base	23/76.7	21/70	21/67.7	0.567	0.445	0.852
3 m	10/33.3^s^	3/10^hs^	7/22.6^hs^	0.028^s^	0.357	0.191
6 m	15/50	10/33.3^hs^	6/19.4^s^	0.197	0.011^s^	0.221
12 m	12/40^s^	16/53.3	12/38.7^s^	0.309	0.920	0.259
***F. nucleatum***						
Base	29/96.7	30/100	29/93.5	0.321	0.581	0.162
3 m	27/90	22/73.3^s^	26/83.9	0.098	0.487	0.323
6 m	28/93.3	23/76.7^s^	21/67.7^s^	0.073	0.011^s^	0.445
12 m	29/96.7	27/90	27/87.1	0.310	0.179	0.727
***P. micra***						
Base	27/90	29/96.7^s^	26/83.9^s^	0.309	0.487	0.096
3 m	24/80	17/56.7^s^	14/45.2^hs^	0.053	0.004^s^	0.377
6 m	26/86.7	19/63.3^s^	13/41.9	0.037^s^	< 0.0001^hs^	0.097
12 m	22/73.3	23/76.7	21/67.7	0.770	0.639	0.445
***C. rectus***						
Base	27/90	25/83.3	28/90.3	0.456	0.967	0.427
3 m	24/80	16/53.3^s^	17/54.8^hs^	0.029^s^	0.037^s^	0.908
6 m	25/83.3	23/76.7	18/58.1^s^	0.527	0.031^s^	0.126
12 m	26/86.7	22/73.3	24/77.4	0.203	0.356	0.717
***F. allocis***						
Base	27/90	28/93.3	25/80.6	0.647	0.311	0.147
3 m	23/76.7	13/43.3^hs^	15/48.4^s^	0.008^s^	0.022^s^	0.698
6 m	23/76.7	19/63.3^s^	15/48.4^s^	0.267	0.023^s^	0.247
12 m	22/73.3	20/66.7^s^	15/48.4^s^	0.581	0.047^s^	0.154

### Immunomarkers

Compared to baseline, IL-1β was statistically significantly reduced at 3 and 6 months in the two antimicrobial-groups. However, this was maintained up to 12 months only in the 7-day AB (Table 5). Nonetheless, at baseline, statistically significantly higher counts of IL-1β were detected in group C compared to the control group. At 12 months, only MMP-8 was statistically significantly reduced in the 7-day antimicrobial-group. Intergroup comparisons revealed no statistically significant differences between the three groups at 12 months (Table 5).

**Table 5:** Quantitative immunological analysis. Mean levels and group comparisons (one way ANOVA, Bonferroni correction for inter- and intra-group comparisons between different timepoints) of immunological (IL-1β, IL-10, TNF α, MMP8) parameters (mean ± SD) (Group A: Placebo, Group B: 3 days AMX + MET, Group C: 7 days AMX + MET; ^s^statistical significant *p* values; IL: Interleukin; TNF: tumor necrosis factor; MMP: matrix metallo protease; Base: baseline, m: months, Δ: change).

Variables	Group A N = 26	Group B N = 24	Group C N = 25	Group comparisons		
				*p* (A–B)	*p* (A–C)	*p* (B–C)
**IL 1β**						
Base	18.00 ± 18.39	26.57 ± 20.46	34.54 ± 24.48	0.275	0.009^s^	0.322
3 m	9.21 ± 11.71	10.66 ± 12.83^hs^	8.00 ± 12.04^hs^	0.892	0.926	1.000
6 m	11.25 ± 15.96	10.00 ± 10.67^s^	9.32 ± 15.55^hs^	0.947	0.868	1.000
12 m	18.36 ± 20.62	18.01 ± 20.76	14.31 ± 14.47^s^	0.998	0.728	1.000
Δ base-3 m	7.06 ± 21.29	15.75 ± 18.10^s^	28.65 ± 26.30^s^	0.298	0.001^hs^	0.091
Δ base-6 m	7.98 ± 26.23	12.09 ± 19.35^s^	27.21 ± 29.25^s^	0.833	0.016^s^	0.107
Δ base-12 m	− 2.64 ± 25.54	9.03 ± 28.12	21.91 ± 29.59^s^	0.321	0.007^s^	0.320
Δ 3 m–6 m	− 1.80 ± 19.26	0.86 ± 12.60	− 2.22 ± 12.27	0.802	0.994	1.000
Δ 6 m–12 m	− 7.29 ± 27.48	− 10.10 ± 23.82^s^	− 3.23 ± 19.92^s^	0.917	0.833	0.981
**IL-10**						
Base	0.65 ± 1.65	0.29 ± 0.77	0.33 ± 0.96	0.476	0.546	1.000
3 months	2.30 ± 4.39	0.66 ± 1.32	0.80 ± 2.39	0.092	0.145	1.000
6 months	2.29 ± 3.99	0.93 ± 2.75	0.54 ± 2.24	0.243	0.089	1.000
12 m	0.93 ± 3.85	1.06 ± 3.48	2.22 ± 5.06	0.993	0.528	0.989
Δ base-3 m	− 1.62 ± 4.80	− 0.39 ± 1.51	− 0.52 ± 2.01	0.297	0.364	1.000
Δ base-6 m	− 1.61 ± 4.40	− 0.68 ± 2.97	− 0.19 ± 2.26	0.576	0.248	1.000
Δ base-12 m	− 0.11 ± 4.19	− 0.81 ± 3.73	− 1.82 ± 5.34	0.852	0.377	1.000
Δ 3 m–6 m	0.09 ± 6.56	− 0.21 ± 2.89	0.25 ± 2.05	0.964	0.990	1.000
Δ 6 m–12 m	1.89 ± 6.40	0.48 ± 3.75	− 1.25 ± 5.65	0.643	0.121	0.797
**IL-8**						
Base	15.84 ± 18.73	23.48 ± 24.74	30.36 ± 37.26	0.553	0.115	1.000
3 m	16.67 ± 23.13	15.98 ± 17.89	9.36 ± 13.72^s^	0.989	0.308	0.343
6 m	27.09 ± 33.23^s^	15.00 ± 15.07	12.32 ± 14.40^s^	0.130	0.043^s^	1.000
12 m	21.34 ± 23.71	18.18 ± 24.22	22.12 ± 24.39	0.891	0.993	1.000
Δ base-3 m	− 0.47 ± 29.13	6.94 ± 21.67	21.89 ± 38.27^s^	0.353	0.218	0.199
Δ base-6 m	− 13.55 ± 37.03	4.64 ± 26.09	19.00 ± 40.43^s^	0.998	0.177	0.424
Δ base-12 m	− 7.67 ± 28.96	0.46 ± 27.50	8.78 ± 40.19	0.096	0.007^s^	1.000
Δ 3 m–6 m	11.92 ± 38.47	0.34 ± 19.13	− 1.97 ± 19.97	0.213	0.951	1.000
Δ 6 m–12 m	5.57 ± 34.78	− 2.35 ± 25.72	− 11.11 ± 19.96	0.308	0.564	0.808
**MMP 8**						
Base	2471.56 ± 1815.96	3335.60 ± 2596 ± 56	4000.64 ± 2320.63	0.313	0.027^s^	0.494
3 m	1153.20 ± 1313.64^s^	3096.09 ± 4814.24	1475.57 ± 1017.55^hs^	0.038^s^	0.913	0.126
6 m	1758.83 ± 1489.02	2437.04 ± 2862.80	1886.99 ± 1225.48^hs^	0.415	0.967	0.904
12 m	2167.45 ± 1299.59	2013.63 ± 1281.73	1774.55 ± 1260.12^hs^	0.907	0.527	1.000
Δ base-3 m	1299.79 ± 24.03.82^s^	140.72 ± 4063.02	2719.78 ± 2844.77^s^	0.353	0.218	0.009s
Δ base-6 m	654.65 ± 2663.64	704.47 ± 4608.73	2234.41 ± 2454.42^s^	0.998	0.177	0.285
Δ base-12 m	− 110.74 ± 1754.95	1367.94 ± 2912.39^s^	2039.03 ± 2480.36^s^	0.096	0.007^s^	0.999
Δ 3 m–6 m	− 669.59 ± 1867.51^s^	737.49 ± 4668.56	− 422.41 ± 1586.20	0.213	0.951	0.497
Δ 6 m–12 m	− 471.55 ± 1933.76	473.00 ± 3078.88	182.99 ± 866.14	0.308	0.564	1.000

Table 6 depicts the results of the Poisson regression analyses showing that antimicrobials, female gender, smoking status, severe forms periodontitis (≥ 10 sites with PD ≥ 6 mm at baseline) and initial CAL-loss, BOP at baseline, detection of *A. actinomycetemcomitans* and *T. denticola* at 12 months as well as initial detection if IL-1β statistically significantly influenced the residual number of deep sites at 12 months (*p* < 0.05).

**Table 6:** Regression analysis for factors influencing the residual number of deep periodontal pockets. Factors influencing the number of residual deep sites (PD ≥ 6 mm) at 12 months as related to placebo (Group A: Placebo, Group B: 3 days AMX + MET, Group C: 7 days AMX + MET). (Poisson regression analysis; PD: pocket depth, CAL: clinical attachment level, BOP: bleeding on probing, GBI: gingival bleeding index^70^, FMPS: full-mouth plaque score after O’Leary^66^, *A.a.: Aggregatibacter actinomycetemcomitans; T.d.: Treponema denticola; P.g.: Porphyromonas gingivalis; F.a.: Filifactor alocis*; IL-1β = Interleukin-1Beta; ^s^statistical significant *p* values. Reference categories are: Group A, Male, Non-Smoker (Exp. Coefficient equals)).

Variables	Exp. coefficient	95% CI	*p* value
Group B (AB for 3 days)	0.24	0.14–0.41	< 0.001^s^
Group C (AB for 7 days)	0.53	0.32–0.87	0.012^s^
Gender	1.39	0.99–1.95	0.052
Smoker	1.36	0.23–0.54	< 0.001^s^
≥ 10 sites with PD ≥ 6 mm at baseline	0.55	0.30–0.99	0.046^s^
BOP baseline	0.21	0.11–0.39	< 0.001^s^
BOP at 12 m	3.30	0.71–15.30	0.126
GBI baseline	1.04	0.98–1.09	0.150
GBI at 12 m	1.46	0.37–5.68	0.585
FMPS baseline	1.02	0.98–1.05	0.241
FMPS at 12 m	0.73	0.21–2.51	0.619
Mean PD baseline	1.01	0.67–1.51	0.946
Mean CAL baseline	1.41	1.17–1.69	< 0.001^s^
*A.a* positive at baseline	1.04	0.73–1.47	0.827
*A.a* positive at 12 m	1.05	0.71–1.55	0.797
*T.d*. positive at baseline	1.01	0.40–2.56	0.974
*T.d.* positive at 12 m	2.17	1.32–3.56	0.002^s^
*P.g*. positive at baseline	0.82	0.43–1.58	0.566
*P.g*. positive at 12 m	1.06	0.67–1.68	0.797
*F.a*. positive at baseline	1.33	0.76–2.29	0.287
*F.a*. positive at 12 m	1.37	0.85–2.22	0.186
IL-1β positive at baseline	1.01	1.00–1.03	0.002^s^
IL-1β positive at 12 m	0.99	0.98–1.01	0.158

## Discussion

The present analyses have evaluated the effect of two systemic antibiotic protocols adjunctive to SD over SD alone on the microbial and inflammatory parameters. Furthermore, the clinical efficacy in terms of pocket closure (PD < 4 mm) and residual deep sites (PD ≥ 6 mm) at 12 months as related to the presence/absence of *P. gingivalis, T. denticola* and *A. actinomycetemcomitans* prior to treatment or at follow-ups was also assessed.

At 6 months, all three treatment protocols succeeded in reducing significantly the counts of *P. gingivalis, T. forsythia, T. denticola, P. micra* and *F. allocis.* However, these substantial bacterial load reductions were maintained up to 12 months in all three groups only for *P. gingivalis, T. forsythia* and *F. alocis.* The pathogens *T. denticola, P. micra* and *F. nucleatum* were still statistically significantly reduced after 1 year only in the two antimicrobial-groups. Moreover, the quantitative reductions of the keystone pathogen *P. gingivalis* were statistically significantly higher at all timepoints in the two antimicrobial-groups as compared to placebo. Considering the fact that the baseline plaque-scores did not differ statistically significantly between the three treatment groups^41^, the better microbial outcomes for the antimicrobial-groups may probably be attributed to the systemic intake of antimicrobials and not to a supra- and subgingival manual biofilm reduction. These results corroborate to those of other studies, where AMX + MET led to statistically significantly higher reductions of the bacterial counts of *P. gingivalis*, *T. forsythia* or *T. denticola* after 3, 6 and/or 12 months^18,23,52–55^.

Contrary to the findings of other research groups^54,55^, *A. actinomycetemcomitans* could not be significantly reduced in any of the groups at 12 months. This may be related to differences in detection methods (i.e. culturing techniques, checkerboard DNA-DNA hybridisation vs. rtPCR)^55^. However, significant quantitative reductions of *A. actinomycetemcomitans* were observed at 3 and 6 months respectively only in the AB groups, being in line with reports of other authors^23,53,56^. Moreover, confirming our results, Eick et al.^52^, showed no statistically significant reduction of *A. actinomycetemcomitans* after 3–6 months after SD with systemic AMX + MET (each 500 mg 3TID for 7 days) neither in patients with chronic nor aggressive periodontitis.

At 12 months, antimicrobials were not able to supress *A. actinomycetemcomitans* or *P. gingivalis* below the detection limit. Almost half of the subjects initially positive on *P. gingivalis* were still positive at 12 months in the two antimicrobial-groups, while 82% of the patients in the placebo group were still positive. The discrepancy between the groups was smaller for *A. actinomycetemcomitans*. These results are in line with those of Eick et al.^52^, showing at 3–6 months post-therapeutically statistically significant reductions of several periodontal pathogens (i.e. *P. gingivalis, T. forsythia, T. denticola, P. intermedia*, e.t.c.) except for *A. actinomycetemcomitans*. Despite the fact that statistically significant reductions were detected for these pathogens, there were still non-neglectable percentages of patients harbouring these bacteria^52^. This corroborates with detection frequencies observed in our study. Moreover, previous reports from other authors have reported that *A. actinomycetemcomitans* had not been completely eliminated by subgingival debridement^6^.

Another explanation would be that, in light of the worldwide indiscriminate use of antimicrobials and implicitly increasing antibiotic resistance^44^, patients may harbour nowadays more resistant strains of these periodontal pathogens as compared to subjects in earlier studies. It has been shown that the reservoir for antibiotic-resistant genes has been increasing over the past century^12,45^. This fact adds on to the capacity of the oral microbiota to transfer antimicrobial resistance^46–49^. For example, *Streptococcus cristaceus* has acquired in vivo doxycycline resistance from a resistant strain of *Streptococcus oralis*, both isolated from the subgingival biofilm of patients under doxycycline therapy^50^. So far, no such direct evidence exists for bacteria of the red complex or *A. actinomycetemcomitans*, but it is very likely that these bacteria as well may have acquired in time resistance to commonly used antimicrobials. Additionally, other authors have already reported high percentages (74.2%) of patients with periodontal pathogens resistant to at least one of the most commonly used antimicrobials in periodontal treatment^57^.

Interestingly, several patients initially negative for *A. actinomycetemcomitans* became positive at 12 months. This may be attributed to the nature of this pathogen being a facultative aerobic bacterium. Pooled samples of subgingival plaque were taken from the deepest site in each quadrant, where most probably anaerobic pathogens were present in higher proportions. After therapy, those sites reduced their depths, resulting in shallow pockets with more aerobic conditions, making thus the detection of *A. actinomycetemcomitans* more probable. Additionally, the sampling method may also have had an impact on the microbiological results, which may possibly reflect an artefact. With the employed sampling strategy, only the chosen deep sites are being examined and their results are being extrapolated on the entire mouth. It is well known that high levels and proportions of periodontal pathogens may also be present in shallow gingival crevices^58^ which had not been assessed. Since *A. actinomycetemcomitans* is facultative anaerobic, it may have as well been present in more shallow sites as opposed to those here analysed (PD ≥ 6 mm), thus leading to false negative results. Another possibility would be the reemergence of *A. actinomycetemcomitans* from other anatomical areas in the oro-pharynx. Data show that *A. actinomycetemcomitans* is presents even in periodontally healthy patients in high numbers on the cheek mucosa (62%), in saliva (59%) and tonsils (41%)^59^. For *P. gingivalis*, the majority of the patients in the placebo group remained positive at 12 months (83%, Table1), while about 50% of the patients in the two antimicrobial-groups became negative; thus, statistically significantly fewer patients in the AB groups harboured this pathogen at 12 months as compared to placebo. These results are in line with those of Rooney et al. who obtained a rare recovery of *A. actinomycetemcomitans* and *P. gingivalis*^19^. Additionally, data from a recent systematic review^60^ indicate comparable percental reductions in the subjects positive on *P. gingivalis* and *A. actinomycetemcomitans*: *P. gingivalis* difference for percental detection rates between test and control group in Dakic et al.: 28% (3 months), 32% (6 months), 34% (12 months) versus our study: 20–40% (3, 6, 12 months); respectively for *A. actinomycetemcomitans* in Dakic et al.: 30% (3 months), 25% (6 months) with no difference between the groups at 12 months^60^. Comparable results were seen in our study: 10–40% difference between the groups at 3 and 6 months, with a slight (10%) to no difference at 12 months between placebo and the two antimicrobial-groups.

From a clinical point of view, statistically significantly fewer residual deep sites (PD ≥ 6 mm) and more sites reached pocked closure at 12 months in the 3-day antimicrobial-group as opposed to placebo. Interestingly, this could not be observed for the 7-day AB-protocol. Analysing the impact of *A. actinomycetemcomitans* or *P. gingivalis* on these clinical parameters, only patients initially negative for *A. actinomycetemcomitans* and initially positive for *P. gingivalis* exhibited at 12 months significantly less residual deep sites as opposed to placebo; nonetheless, at 6 months, *A. actinomycetemcomitans* positive patients showed in both antimicrobial-groups statistically significantly fewer residual sites compared to placebo. This underlines the fact that antimicrobials had a statistically significant positive effect also on patients negative for this periodontal pathogen compared to those not receiving the medication, and is in line with reports of other studies^23^. These results are further supported by the recent findings of a systematic review that stressed out the efficacy of AMX + MET in improving clinical outcomes irrespective of the initial detection of *A. actinomycetemcomitans*^15^.

Moreover, the results of the present regression analysis showed that neither presence of *A. actinomycetemcomitans* and *P. gingivalis* at baseline nor at 12 months were associated with a higher risk for residual deep sites at 12 months (*p* < 0.05). These results corroborate those of Mombelli et al., who have also observed that the presence/absence of *A. actinomycetemcomitans* did not influence the effect of antimicrobials on persisting active sites with PD > 4 mm^56^. Antimicrobials seemed to be highly efficient in both *A. actinomycetemcomitans* positive and negative patients. Additionally, antimicrobial-groups had significantly more sites reaching pocket closure (PD < 4 mm) at 6 months compared to placebo which is in line with Kolakovic et al.^61^, who obtained in a meta-analysis a 4.43 higher chance for pocket closure at 6 months in the AMX + MET group compared to control. Moreover, the results in our study were maintained up to 1 year. Further subgroup analyses based on the presence/absence of selected periodontal pathogens could not show any statistically significant influence on the number of sites reaching pocket closure (*p* > 0.05), despite the fact that the patients receiving placebo had fewer healed sites (PD < 4 mm) as opposed to those receiving systemic medication.

Nonsurgical periodontal therapy with the two antibiotic protocols yielded a decrease in the levels of proinflammatory markers (IL-1β, IL-8, MMP-8) and an increase of the cytokine IL-10, that is associated with periodontal health. Cytokine analysis resulted in a significant reduction of the proinflammatory immunomarker IL-1β at 3 and 6 months compared to baseline only in the two antimicrobial-groups. This could be observed in the 7-day antimicrobial-group also at 12 months, which however, had at baseline significantly higher levels of IL-1β. Additionally, the initial detection of IL-1β was shown to statistically significantly influence the residual number of deep sites at 12 months. The other proinflammatory cytokines (IL-8, MMP-8) showed reductions at 6 and 12 months only in the two antimicrobial-groups. Nonetheless, these reductions did not reach statistical significance except for MMP-8, that was statistically significantly reduced compared to baseline in the 7-day antimicrobial-group; however, MMP-8 intergroup comparisons revealed no significant differences between the three groups at 12 months. These results compare well to those of Jentsch et al., where slightly higher median values for IL-1β and comparable results for MMP-8 were reported for the AMX + MET group at 3 and 12 months respectively^62^.

The present cytokine reductions in the two antimicrobial-groups, may indeed account for the additional general anti-inflammatory effect of systemic antimicrobials, especially since no statistically significant group differences for baseline plaque scores had been detected^41^.

The current study represents a secondary analysis of the microbiological and inflammatory data of a clinically designed RCT^33,41^, which may be a limitation for the power of the current results. Considering the fact that the study was initially designed to find a difference between the control and the test groups for a clinical parameter (5 sites with PD ≥ 6 mm), the presented results and subgroup analyses should be interpreted with caution. Nevertheless, these microbiologic and immunologic data corroborate the primary clinical outcomes of the present trial^33,41^ supporting the finding that both antibiotic protocols adjunctive to mechanical debridement elicit better clinical, microbiological and immunological effects in severe forms of chronic periodontitis (stage III-IV, grade B). It is important to emphasize that the study was designed to evaluate the efficacy of the two antibiotic regimes adjunctive to non-surgical periodontal therapy compared to mechanical debridement alone. The protocol had not been designed to determine the equivalence or non-inferiority of the use of a 3-day over the 7-day antibiotic protocol adjunctive to mechanical debridement and thus no clear clinical recommendations related to the preferential use of a 3-day AB course over a 7-day one can be made. Moreover, considering the global increase in antimicrobial resistance, antimicrobial usage adjunctive to non-surgical periodontal therapy should be carefully taken prescribed only in selected patients with generalised severe forms of periodontitis that may really profit from it^63^.

In conclusion, the present data indicate that the systemic use of both 3- and 7- day AMX + MET adjunctive to non-surgical periodontal therapy may induce superior microbiological outcomes compared to subgingival debridement alone. The two investigated antibiotic protocols led to comparable microbiological and inflammatory results.

## Methods

This study was a prospective, randomized, placebo controlled, double-masked clinical trial. Following hypothesis was tested: “the systemic use of AMX and MET administered for 3 or 7 days as adjunct to SD leads to superior clinical results compared with SD alone”^33^. The clinical results after 6 and 12 months were previously published^41^. The study protocol (according to the Declaration of Helsinki 1964, revision 2008) was approved by the Ethical Committee of the Faculty of Medicine and Pharmacy of Cluj-Napoca (Trial registration number #514/09.01.2012, registration date: 20.02.2012) and was registered in the ISRCTN registry (trial registration number: ISRCTN17605083, registration date: 16.6.2015, https://www.isrctn.com/ISRCTN17605083).

### Subjects and clinical protocol

The clinical protocol was described in detail previously^33^. Briefly, 102 subjects seeking dental treatment at the University clinic (University Cluj-Napoca) were included and treated between January 2012 and January 2014. Included patients were over 30 years old, had a minimum of 12 natural teeth in the oral cavity and clinical (at least two sites/quadrant with PD ≥ 6 mm) and radiographic signs of generalized severe chronic periodontitis^64^ (generalized stages III–IV, grade B periodontitis)^65^**,** good level of oral hygiene [Full-mouth plaque-scores (FMPS) ≤ 25% prior to SD^66^], systemically healthy. Subjects smoking at least 10 cigarettes/day for the last 5 years were defined as smokers^67^. Patients with systemic/local antibiotic therapy within the preceding 3 months, or any type of medication with a possible influence on the periodontium, pregnant/lactating patients or those who had non-surgical periodontal therapy within the previous 12 months were excluded. Informed written consent to participate in the study was obtained from all participants.

**Figure 1:**
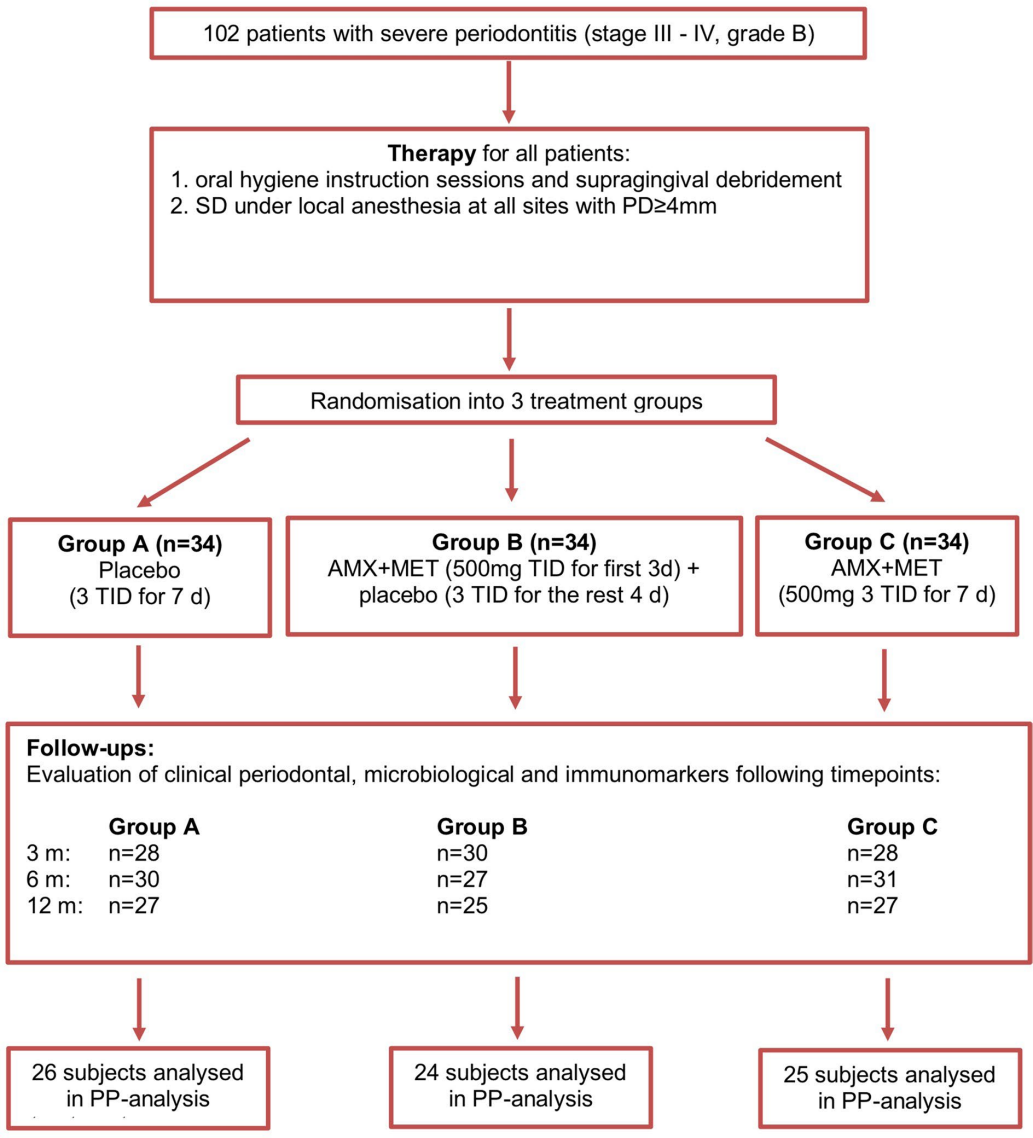
Flowchart of the study. Included subjects were instructed for adequate oral hygiene and nonsurgically treated (SD: subgingival debridement). Immediately after SD, patients were randomised to medication (AMX-amoxicillin, MET-metronidazole, TID-times per day). Patients were reevaluated at 3, 6 and 12 months when clinical periodontal parameters, microbiological and immunological outcomes were determined. Appropriate subject numbers at the follow-ups are given. The final number of subjects included in the per-protocol analysis (PP-analysis) is provided.

Medical history, clinical periodontal parameters (at 6 sites/tooth: PD, CAL), bleeding on probing (BOP) and FMPS^66^ were recorded before and 3, 6 and 12 months after non-surgical periodontal therapy. Additionally, gingival crevicular fluid (GCF) and subgingival biofilm samples from the deepest pocket in each quadrant were taken at the same timepoints. All recordings, sampling and oral hygiene instructions were performed by one examiner (RJ) blinded to treatment allocation. SD (i.e. elimination of the subgingival calculus and biofilm) was performed within 24h^63,68^ was performed within 24 h at all sites with PD ≥ 4 mm by one experienced periodontist (RC), blinded to group allocation^33,41^. A computer-generated block-randomisation list divided the patients afterwards in three treatment groups:

Group A: SD + placebo [placebo three times/day (TID) for 7 days].
Group B: SD + systemic AMX + MET (both 500 mg 3TID for first 3 days, placebo 3TID for the rest 4 days).
Group C: SD + systemic AMX + MET (both 500 mg 3TID for 7 days) (Fig. 1).

Patient allocation was performed after SD by another clinician (RT) [for details regarding the bottle pills please see^33^].

At the follow-up appointments (3, 6, 12 months), the above mentioned parameters were recorded and only supragingival calculus was removed. Any residual periodontal pockets (pockets with PD = 4 mm + BOP, or PD ≤ 5 mm) were not re-instrumented.

### GCF and microbial sampling and analysis

In each quadrant, the site with the deepest PD was selected for GCF and microbial sampling. After isolation with cotton rolls and careful removal of supragingival plaque with cotton pellets, a standard paper-strip (Periopaper, Oraflow, USA) was placed at the entrance of the periodontal pocket for 30 s. After removal of the paper strip, a sterile paper-point was inserted for 30 s into the gingival crevice until mild resistance was felt. The four paper strips and four paper points obtained at one timepoint were each pooled into a sterile empty transportation container. GCF-samples were stored at − 70 °C and microbial samples at − 20 °C until assayed.

The host-derived biomarkers IL-1β, IL-10, IL-8 and MMP-8 were determined by commercially available ELISA-kits (R&D Systems Europe Ltd., Abingdon, UK) according to the manufacturer’s instruction. The detection levels of 2 pg/sample for IL-1β, IL-10 and IL-8 and 0.1 ng/sample for MMP-8. Samples were eluted over night at 4 °C into 750bµl phosphate-buffered saline (PBS, SigmaAldrich, St. Louis, MO, USA) and then centrifuged at 400 g for 2 min. For ELISA, 100 µl aliquots of the supernatant were removed and used. The detection levels of the used test kits were 5 pg/sample (IL1β, IL-8, IL-10, MMP-8)^69^.

Real-time polymerase-chain-reaction (rtPCR) was used to detect the periodontal pathogens *A. actinomycetemcomitans, P. gingivalis, Tannerella forsythia, Treponema denticola, Parvimonas micra, Fusobacterium nucleatum, Campylobacter rectus* and *Filifactor allocis*. Bacterial DNA was extracted with Chelex 100 (Bio Rad Laboratories, Inc., Berkeley, CA, USA) and rt PCR was carried out (ABI 7500 rtPCR System, Applied Biosystems, Foster City, CA, USA). rtPCR amplifications were carried out using a 25 µl reaction volume [2.5 µl DNA template and 22.5 µl substrate mixture: 1.25 µl of each primer, 12.5 µl GoTaq qPCR Master Mix, 2 × Promega Corporation (Madison, WI, USA), 5 µl nucleic acid free H_2_O]. Each batch of specimens contained negative (H_2_O) and positive controls (2.5 µl genomic DNA of the respective reference strains in different concentrations). The cycling steps consisted of an initial denaturation step at 95 °C for 2 min, 45 cycles at 95 °C for 15 s and at 60 °C for 60 s. The specificity of the amplification was assayed using melting curves and the detection level was 100 bacteria/sample^69^.

### Statistical analyses

Statistical analyses were performed by an experienced professional statistician (CH) using the statistical software program (SPSS statistics 21, IBM, NY, USA). The present study was initially designed to find a difference between the control and the test groups for clinical parameters as the primary outcome^33,41^. Shortly, the statistical unit was the patient and the primary outcome variable of the study was the difference in the number of sites per patient with PD ≥ 6 mm calculated between baseline and 12 months^41^. The power of the study was calculated for a difference of at least 5 sites with a PD ≥ 6 mm (standard deviation of 6 sites) between both AB and placebo groups. A study power of 92% for a statistical significance level of 0.05 was achieved for 30 subjects/ group; considering an attrition of 13%, 34 subjects/group were initially included.

For the clinical variables, intra-group clinical comparisons between the follow-ups were analysed by means of paired *t*-Test and Wilcoxon Signed Ranks Test. Comparisons between the groups at the various timepoints were performed adjusting for baseline values and smoking using ANCOVA and Bonferroni corrections.

Quantitative analysis of the microorganisms and inflammatory markers were first checked for normal distribution using Kolmogorov Smirnov test. Depending on these results, intra-group comparisons between the various timepoints were performed by means of paired t-Test and Wilcoxon Signed Ranks Test. For qualitative changes of the immunomarkers, the Chi-square test was used, while for microbial changes McNemar Test was implemented. Inter-group comparisons were performed by means of ANCOVA, Mann–Whitney *U* and Kruskal–Wallis tests. Frequencies/number of patients positive for the microorganisms and immunomarkers were determined and intragroup comparisons were performed using McNemar test.

The residual number of sites with PD ≥ 6 mm as well as the number of sites reaching the clinical endpoint of pocket closure (< 4 mm) at 12 months were specifically determined for patients being positive/negative at baseline or at 6- and 12 months for the pathogens *A. actinomycetemcomitans, P. gingivalis and T. denticola;* group comparisons were performed using ANCOVA.

Finally, a Poisson regression analyses was conducted to determine the influence of the 3- or 7-day antimicrobial (AB) protocol, gender, smoking, BOP and FMPS at baseline and 12 months, initial mean PD and CAL, disease severity (≥ 10 sites with PD ≥ 6 mm), detection of *A. actinomycetemcomitans, P. gingivalis, T. denticola, F. allocis* and IL-1β at baseline on the residual number of deep sites (PD ≥ 6 mm) at 12 months.

## Data Availability

Study protocol data are available https://www.isrctn.com/ISRCTN17605083. Data results are available from ralucacosgrea@gmail.com.

## Code availability

The study has been registered in the ISRCTN registry (registration date 16.06.2015, registration number: study ID ISRCTN17605083) and is available on the website http://www.isrctn.com/ISRCTN17605083.
